# Incidence of maternal near-miss in Kenya in 2018: findings from a nationally representative cross-sectional study in 54 referral hospitals

**DOI:** 10.1038/s41598-020-72144-x

**Published:** 2020-09-16

**Authors:** Onikepe Owolabi, Taylor Riley, Kenneth Juma, Michael Mutua, Zoe H. Pleasure, Joshua Amo-Adjei, Martin Bangha

**Affiliations:** 1 Research Division, Guttmacher Institute, 125 Maiden Lane, 7th Floor, New York, NY 10038 USA; 2 Population Dynamics, Sexual and Reproductive Health Unit, African Population Health and Research Center, Manga Close, Nairobi, Kenya; 3 University of Cape Coast, Cape Coast, Ghana

**Keywords:** Public health, Epidemiology, Epidemiology

## Abstract

Although the Kenyan government has made efforts to invest in maternal health over the past 15 years, there is no evidence of decline in maternal mortality. To provide necessary evidence to inform maternal health care provision, we conducted a nationally representative study to describe the incidence and causes of maternal near-miss (MNM), and the quality of obstetric care in referral hospitals in Kenya. We collected data from 54 referral hospitals in 27 counties. Individuals admitted with potentially life-threatening conditions (using World Health Organization criteria) in pregnancy, childbirth or puerperium over a three month study period were eligible for inclusion in our study. All cases of severe maternal outcome (SMO, MNM cases and deaths) were prospectively identified, and after consent, included in the study. The national annual incidence of MNM was 7.2 per 1,000 live births and the intra-hospital maternal mortality ratio was 36.2 per 100,000 live births. The major causes of SMOs were postpartum haemorrhage and severe pre-eclampsia/eclampsia. However, only 77% of women with severe preeclampsia/eclampsia received magnesium sulphate and 67% with antepartum haemorrhage who needed blood received it. To reduce the burden of SMOs in Kenya, there is need for timely management of complications and improved access to essential emergency obstetric care interventions.

## Introduction

Globally, there is increased recognition of the importance of using severe maternal morbidity to evaluate progress towards improving obstetric outcomes^1,2^. Thus, maternal near-miss (MNM), defined by the World Health Organization (WHO)^3^ as “a woman who nearly died but survived a complication that occurred during pregnancy, childbirth or within 42 days of termination of pregnancy”, has been used more frequently as a standardized outcome to evaluate and improve the quality of obstetric care^4^. Using MNM as an endpoint is valuable because its causes are similar to those of maternal mortality, and MNM events occur more frequently than maternal deaths (MD)^5^, leaving open the potential for individuals to be interviewed. This gives MNM studies greater power to identify factors associated with adverse maternal outcomes^6^, and allows us the opportunity to generate recommendations to address them at subnational and individual health facility levels^7^. However, studies that have attempted to apply the WHO MNM criteria in low-income settings have critiqued it for having lower applicability in these contexts. Hence, a number of adaptations to the criteria have been recommended to avoid under-reporting of cases^8,9^.

In Kenya, progress on reducing maternal mortality appears to be slow despite efforts by the government within the last 15 years such as: introducing a reproductive health voucher program subsidizing care in 2006^10^, and provision of free maternity services in public facilities in 2013^11^. The maternal mortality rate (MMR) in the 2014 Demographic and Health Survey (DHS) remained high at 362 deaths per 100,000 live births^12^, and one study evaluating the impact of free maternity services found an increase in utilization but no significant decline in national MMR^11^.

With the challenges of using MMR as a tracking indicator and the relative rarity of maternal deaths, collecting nationally representative data on MNM could provide more detailed information for Kenyan policy makers to better evaluate the healthcare system and identify approaches to achieve national targets. The objective of our study was to describe the incidence and cause distribution of MNM in Kenya and the quality of clinical management of these complications. We also explored the utility of the WHO near-miss criteria in the Kenyan context.

## Methods

### Design, setting and population

We conducted a cross-sectional study within a nationally representative sample of public and private referral-level facilities in Kenya sub-county, county and national hospitals within a three-month period between February and May 2018. This period followed an extended national health workers strike by doctors and then nurses in Kenya which ended in November 2017, and some facilities started later than others due to administrative bottlenecks^13^. All county (n = 16) and national (n = 2) hospitals were eligible for participation. We generated a simple random sample, stratified by region, of all sub-county hospitals (n = 424) and 46 were selected to participate. Selected facilities that declined to participate, or that were non-functional at the time of the survey were replaced with similar-level facilities drawn from a replacement list generated before the study commenced. Fifty-four facilities participated in the study, with a response rate of 86% (Supplementary Table S1 shows the sampling and response rates for facilities and patients).

All patients of reproductive age admitted with a potentially life-threatening condition (PLTC), or as an MNM, or an MD that occurred in the facility during pregnancy, delivery or within 42 days of delivery or termination of pregnancy were eligible for inclusion. PLTCs are defined as “an extensive category of clinical conditions, including diseases that can threaten a woman’s life during pregnancy and labour and after termination of pregnancy^3^.” PLTCS may recover from their conditions with clinical management or progress to become MNMs, which may similarly recover with clinical care or result in MDs. MD was defined according to the International Classification of Disease (ICD-10)^14^. Severe maternal outcomes (SMO) included all MNMs and MDs. Informed consent was sought from eligible patients when they were treated and in a clinically stable condition before discharge. Trained study clinicians extracted individual-level data from patient files for patients who consented to participate in the study. One patient who experienced a MNM event did not consent to participate in the study (0.3%) and we were unable to obtain consent for participation for seven patients who died (29%) (data not shown).

We used two methods for defining MNM criteria: the WHO operational definitions, based on organ failure, and an adaptation of these operational definitions for the Kenyan context (see Supplementary Table S3 for comparison). We included the Kenyan adapted criteria as evidence from other studies on MNM in low- and middle-income countries (LMICs) suggests that the original WHO criteria (particularly the management and laboratory-based criteria) often has limited applicability in such contexts^8^. We however retained all the original WHO criteria in our instrument to allow for comparisons with studies from other contexts. Questions for the Kenyan adaptation were added to questions from the published WHO MNM surveillance and assessment tool, which we used to develop our data collection tool^3^. These adaptations were selected based on previous studies attempting to validate the criteria of the WHO near-miss approach in other LMICs and with input from the clinicians participating in the study^15,16^. All MNM indicators were defined according to the WHO near-miss manual^3^. For some conditions within the WHO MNM organ dysfunction categories (shock, abnormal liver enzymes, and massive blood transfusion), we collected detailed information on the clinical signs and symptoms used to diagnose each condition.

### Data collection

Each participating facility identified one study clinician, such as a medical doctor, clinical officer or nurse, who was trained to conduct face-to-face interviews and extract data from medical records. The national hospitals and county hospitals with expected higher caseloads had two to three interviewers. All facility interviewers participated in a two-day training on the study procedures, and piloted the tool in a sub-county hospital in Nairobi. The facility interviewers visited the obstetrics wards, delivery rooms, emergency rooms and intensive care units daily to identify eligible patients. Each eligible patient admitted (except MDs) was first approached by their health care provider who informed them about the study and asked if the study team could speak with them. If they agreed to speak to the study team and was in a stable condition, the facility interviewer further explained the study and obtained their written consent to participate. Consent included permission to interview them, their health care provider and to review their medical records. Upon receiving each informed consent to participate in the study, the study clinicians interviewed the patient’s health care provider, reviewed their health records to extract information about their clinical condition using the study tool, and interviewed them to collect any information not recorded in their health records. We also extracted individual level data for patients who came in with a complication, died and had consented to participate in the study before they died. Patients who did not consent (either because they did not give consent or could not provide consent before they died), and those who were dead on admission were recorded in the monthly caseloads and included in the intra-hospital MMR and mortality index, but their individual data was not analysed.

The study team provided regular oversight of the study process to assess quality and completeness of data collection. To minimize the number of missed cases, we created a daily log for the study clinicians to track all patients perceived to have serious conditions across the relevant wards in each facility. Thereafter, the study clinicians reviewed the medical records of each tracked case to determine if the patient was admitted with or developed any PLTCs that would make them eligible for the study. We also produced a visual guide of PLTCs to remind interviewers how to determine study eligibility.

Before data entry, a medical doctor performed validity crosschecks of questionnaires for clinical inconsistencies or missing data. Double data entry was done for 10% of data collection forms and inconsistency checks programmed in the statistical software to flag any potential errors. If errors were found, the study team followed up with the facility interviewers to verify or obtain the correct information from the patient’s medical records related to these discrepancies.

We used a structured data collection form to obtain the total number of deliveries, live births, gynaecological admissions, post-abortion care admissions, and MDs occurring during each month of the study period from each facility’s Health Management Information System (HMIS).

### Outcome measures

- *Severe maternal outcome ratio (SMOR)* The number of patients with life-threatening conditions (i.e. maternal death and near-misses) per 1,000 live births· [SMOR = (MNM + MD)/LB].
- *Maternal near-miss ratio (MNMR)* the number of maternal near-miss cases per 1,000 live births (MNMR = MNM/LB).
- *Intra-hospital MMR* The number of maternal death that occurred in the hospital per 100,000 live births.
- *Mortality index (MI)* The number of maternal deaths divided by the number of patients with life-threatening conditions (i.e. maternal near-misses plus maternal deaths) expressed as a percentage [MI = MD/ (MNM + MD)].
- *Maternal near-miss mortality ratio (MNM: 1 MD)* The ratio between maternal near-miss cases and maternal deaths.

### Analysis

We conducted descriptive analyses of study participants characteristics, underlying and contributory causes of severe morbidity, and the distribution of organ dysfunction, by type of SMO. National estimates of MNM were generated using the adapted Kenyan definition. The number of MNM cases was annualized from the three-month study period and weighted for study design to obtain a national annual incidence of MNM for 2018. We estimated the SMO ratio (SMOR), intra-hospital MMR, MNM ratio (MNMR), and other MNM indicators at the national and regional levels. We also described the corresponding standards of care for each complication to assess the quality of care provided using the WHO MNM guidelines. We compared the number of MNM generated for each organ dysfunction category and their criteria using our adapted criteria and the WHO criteria. We also examined if the WHO approach of asking clinicians to select checkboxes to indicate a diagnosis of some MNM conditions aligned with the clinical definitions WHO provided for these conditions. To do this, we compared the selection of diagnoses using just the checkbox to a diagnosis generated in statistical software using the constellation of clinical signs and symptoms WHO requires to detect the condition diagnosed. Data analysis was conducted using Stata Version 15.1^17^.

## Ethical approval

The Kenya Medical Research Institute (KEMRI) institutional review board reviewed and approved this study on December 20, 2017 (protocol ID: KEMRI/RES/7/3/1), African Population and Health Research Centre (APHRC) received approval from the National Commission for Science Technology and Innovation (NACOSTI) on March 28, 2018 (Approval Ref: NACOSTI/P/18/46177/21949), and Guttmacher Institute institutional review board on September 26, 2016 (DHHS identifier IRB00002197). We did not obtain parental/legal guardian consent in this study but received a waiver for it from all IRBs concerned because in Kenya all individuals under the age of 18 in our sample were previously pregnant, and are therefore considered emancipated minors under Kenyan law. All research methods were performed in accordance with relevant guidelines/regulations.

## Results

### Primary outcome measures

During the period of data collection, there were 36,162 (unweighted) live births, 318 (unweighted) patients with PLTCs who did not experience a MNM event or die and 377 (unweighted) cases with an SMO (360 MNM and 17 MDs). At the national level, we estimated an annual intra-hospital MMR of 36.2 per 100,000 live births (0.04%), an MNM ratio of 7.2 per 1,000 live births (0.7%), such that there were 20 MNMs for each MD in hospitals. We also estimated a national mortality index in hospitals of 4.8%. Although most SMOs occurred before admission to a hospital (64%), and the majority of these cases were referred from other facilities (58%), 36% of SMOs occurred whilst patients were admitted (Table 1).

**Table 1:** Estimated annual incidence of maternal near-miss and other severe maternal outcome indicators in Kenya in 2018.

	National
**Annual number of facility caseloads**	
Total number of facility live births	708,459
Total number of facility deliveries	726,158
**Annual number of severe maternal outcomes**	
Number of maternal near-miss cases	5,116
Number of maternal deaths	256
**Overall maternal near-miss indicators**	
Severe maternal outcome ratio (per 1,000 live births)	7.6 (4.93–10.24)
Maternal near-miss incidence ratio (per 1,000 live births)	7.2 (4.57–9.87)
Intra-hospital maternal mortality ratio (per 100,000 live births)	36.2 (13.32–59.00)
Mortality index (MI)^a^	4.8%
Maternal near-miss mortality ratio	20.0
**Hospital access indicators**	
Severe maternal outcome occurred before admission in hospital	64%
Severe maternal outcome occurred during stay in hospital	36%
Severe maternal outcome occurred before admission, referred from other facilities	58%
Mortality index: severe maternal outcome occurred before admission	1.6%

All estimates are weighted.

^a^Mortality index is calculated as: maternal deaths/(maternal near-miss + maternal deaths).

### Characteristics of study population

Table 2 describes the demographic characteristics of patients with PLTCS and SMOs. Whilst the majority of patients with PLTCs that did not become more severe after receiving clinical care, MNM cases and MD were between ages 20–34, 8% of MNM cases and 14% of patients with PLTCs were adolescents aged 15–19. The majority of patients with PLTCs, MNM events and MD received any antenatal care (ANC) from a skilled provider (76%, 66%, and 82%, respectively). The percentage of PLTCs, MNMs and MD with a live birth after this pregnancy who received ANC from a skilled provider (similar to the DHS indicator) were much higher (data not shown, 100%, 92% and 84% respectively). Almost two-thirds of MNM (65%) and less than one-third of MD (32%) experienced their severe obstetric complication before admission to the facility. Whilst approximately a quarter of MNM cases and MD delivered via caesarean section, 20% of MNM cases had a laparotomy for ectopic pregnancy whilst 9% had a spontaneous or induced abortion. About a fifth of patients who experienced a MNM event also experienced a foetal death.

**Table 2:** Characteristics of patients with potentially life-threatening conditions, maternal near-miss events, and maternal death, Kenya 2018.

Characteristics of respondents^a^	Potentially life-threatening conditions (PLTC)^b^		Maternal near-miss (MNM)		Maternal death (MD)^c^	
	N	%	N	%	N	%
**Age**						
15–19	269	14	105	8	–	–
20–24	436	22	367	29	3	7
25–29	465	24	359	28	18	43
30–34	458	23	252	20	15	35
35+	344	17	195	15	6	14
**Number of pregnancies**						
1	471	24	375	29	13	31
2	391	20	201	16	14	33
3	402	20	203	16	5	12
4+	708	36	500	39	10	24
Received any professional antenatal care^d^	1,451	76	835	66	32	82
**Estimated gestational age**						
First trimester	272	16	244	25	–	–
Second trimester	177	11	102	10	1	3
Third trimester	1,228	73	643	65	37	97
**Timing of severe obstetric complication**						
Before admission	1,266	66	813	65	11	32
During hospital stay	647	34	442	35	23	68
**Final mode of delivery**						
Vaginal delivery	460	24	513	40	30	77
Caesarean section	873	45	334	26	9	23
Abortion (spontaneous or induced)	281	14	109	9	–	–
Laparotomy for ectopic pregnancy	179	9	256	20	–	–
Woman discharged or referred still pregnant	151	8	64	5	–	–
**Foetal outcome at birth**						
Alive	1,119	90	656	78	38	97
Dead	122	10	186	22	1	3
Total number of patients in each category of obstetric complications	1972	100	1,279	100	42	100

All estimates are weighted from the three-month study period and include only patients who consented to be interviewed.

PLTC: Professional ANC care—12; Estimated EGA—60; Timing of SMO—18; Final mode of delivery—4; Foetal outcome at birth—115. MNM: Professional ANC care—6; Estimated EGA—96; Timing of SMO—9; Final mode of delivery—2; Foetal outcome at birth—108.

^a^Unweighted missing cases for each characteristic variable.

^b^PLTC does not include patients who were classified as a MNM or MD.

^c^Patients who did not consent and were a maternal death were not included in the maternal death total in this table, but are included in the incidence calculations.

^d^Skilled provider includes-obstetrician/gynaecologist, medical officer, clinical officer, nurse or midwife.

### Major causes of SMO and distribution of organ dysfunction in MNM cases

Obstetric haemorrhage and hypertensive disorders were the most common underlying causes of SMOs (Table 3). About a fifth of all MDs were due to hypertensive disorders and pregnancies with abortive outcomes contributed to 12% of MDs and 9% of MNM. The contribution of other obstetric diseases or complications to MNM (28%) was substantially more than their contribution to MD (2%). Previous caesarean Sect. (16%) was the biggest contributory cause to MNM followed by anaemia (8%). Amongst MNM, hematologic dysfunction was the most frequently identified organ dysfunction (36%) whilst respiratory dysfunction was the most common amongst MDs (93%). The mortality index was highest for respiratory dysfunction (13%) and lowest for renal, uterine, and hepatic dysfunctions (1%, 1% and 2% respectively).

**Table 3:** Distribution of organ dysfunction, underlying causes and contributory conditions among maternal near-miss cases and maternal deaths, Kenya 2018.

Organ dysfunction^a^	Potentially life-threatening conditions		Maternal near-miss		Maternal deaths^d^		Mortality index
	N	%	N	%	N	%	%
Cardiovascular	n/a	n/a	342	27	35	83	9
Respiratory	n/a	n/a	255	20	39	93	13
Renal	n/a	n/a	316	25	2	5	1
Hematologic	n/a	n/a	457	36	21	50	4
Hepatic	n/a	n/a	142	11	3	7	2
Neurologic	n/a	n/a	219	17	14	34	6
Uterine^b^	n/a	n/a	292	23	2	5	1
**Underlying causes**^a^							
Pregnancy with abortive outcome	291	15	112	9	5	12	4
Obstetric haemorrhage	1,181	60	686	54	24	57	3
Hypertensive disorders	442	22	327	26	9	21	3
Pregnancy-related infections	117	6	75	6	2	5	3
Other obstetric disease or complications	783	40	354	28	1	2	0
Medical/surgical/mental disease or complications	2	0	45	4	2	5	4
**Contributory causes/associated conditions**^a^							
Anaemia	234	15	103	8	1	2	1
HIV infection^c^	115	6	31	3	1	4	3
Previous caesarean section	312	16	204	16	1	2	0
Prolonged obstructed labour	552	28	46	4	1	2	2
Total	1,972	100	1,279	100	42	100	

All estimates are weighted from the three-month study period and include only patients who consented to be interviewed.

^a^These categories are not mutually exclusive and can add up to more than 100%.

^b^Includes paralytic ileus (absent bowel sounds in a gaseous distended abdomen), PTE (pulmonary thromboembolism) showing the above respiratory symptoms and signs, hysterectomy following infection or haemorrhage, and laparotomy.

^c^Five patients who died were missing on HIV status so the denominator used to estimate this proportion is smaller than for other contributory causes.

^d^Patients who did not consent and were a maternal death (unweighted n = 7) were not included in the maternal death total in this table, but are included in the incidence calculations.

### Quality of definitive clinical care provided for major obstetric conditions

Table 4 shows the standards and processes of care provided for each of the major direct obstetric complications. Of the patients with severe pre-eclampsia/eclampsia, 77% received magnesium sulphate, while 93% of patients with postpartum haemorrhage (PPH) received oxytocin or ergometrine. Although blood was requested for 84% of patients with antepartum haemorrhage (APH), only 67% of those needing blood received transfusions. Amongst patients with a ruptured uterus, 44% had a laparotomy within three hours of admission. Almost all patients with a caesarean section and those with severe infection or sepsis received parenteral antibiotics for prophylaxis and as treatment respectively (98% and 99%).

**Table 4:** Process and outcome indicators for selected obstetric complications, Kenya 2018.

Interventions, by selected obstetric complications	Number and percentage of patients	
	N	%
**Antepartum haemorrhage (APH)**		
Target population: patients with APH	226	7
Requested blood	191	84
Blood given (out of those with blood requested)	109	67
IV fluids given	224	99
Cases with SMO^a^	96	43
Mortality^b^	–	–
**Postpartum haemorrhage (PPH)**		
Target population: patients with PPH	1755	53
Oxytocin, ergometrine, oxytocin/ergometrine	1597	93
Misoprostol	740	45
Other uterotonic	57	4
Tranexamic acid	450	27
Removal of retained products	704	43
Balloon or condom tamponade	97	6
Artery ligation	32	2
Hysterectomy	70	4
Uterine packing, uterine massage	821	50
Repair of tears	338	21
Cases with SMO	670	38
Mortality	24	1
**Severe pre-eclampsia/eclampsia**		
Target population: patients with severe pre-eclampsia/eclampsia	778	24
Magnesium sulphate	580	77
Other anticonvulsant	116	16
Cases with SMO	137	67
Mortality	9	1
**Severe infections/sepsis**		
Target population: patients with sepsis/severe infections	194	6
Parenteral antibiotics	192	99
Cases with SMO	77	40
Mortality	2	1
**Caesarean section related complications**		
Target population: patients undergoing caesarean	1,216	37
Prophylactic antibiotic	1,181	98
Cases with SMO	343	28
Mortality	9	1
**Ruptured uterus**		
Target population: patients with ruptured uterus	74	2
Laparotomy after 3 h of hospital stay	33	44
Cases with SMO	53	71
Mortality	–	–

All estimates are weighted from the three-month study period and include only patients who consented to be interviewed. Obstetric complications are not mutually exclusive; a patient can have more than one type of complication.

^a^SMO = severe maternal outcome includes maternal near-miss and maternal death.

^b^Patients who did not consent and were a maternal death (unweighted n = 7) were not included in the maternal death total in this table, but are included in the incidence calculations.

### Near-miss indicators by Kenyan sub-region

Supplementary Table S2 describes the MNM indicators by region. The MNMR and SMOR ranged from 4.3 per 1,000 live births (0.4%) and 5.1 per 1,000 live births respectively (0.5%) in Eastern region to 9.4 per 1,000 live births (0.9%) and 9.7 per 1,000 live births (1%) respectively in the Rift Valley. Mortality indices varied greatly across regions from 0.6% in Nyanza and Western region to 15.6% in the Eastern region. Similarly, the intra-hospital MMR was highest for hospitals in the Eastern region. Rift Valley recorded the highest number of SMOs occurring before admission to a hospital (73%) whilst Nyanza and Western had the highest number of SMOs occurring during their hospital stay (55%).

### Comparison between case yield from WHO near-miss criteria and the Kenyan adaptation

While the WHO criteria identified 250 MNM cases, the Kenya-adapted criteria identified 360 cases—about 1.4 times the WHO criteria. Supplementary Table S3 shows the number of events in each subcategory of each organ/system. Using the Kenyan criteria for transfusion of two or greater units of blood and blood products in cases of severe anaemia in pregnancy, we identified 60 MNM cases compared with 42 using the WHO criteria of five or more units of blood. However, all the patients missed within the WHO transfusion criteria would have been identified as MNM by the other WHO criteria.

We explored which criteria within the Kenyan adaptation identified over 5% of additional MNM cases that would have been missed using the WHO criteria. They include our definition of shock (10%) and undergoing a laparotomy (7%) other than a caesarean section.

## Discussion

Our findings suggest that Kenya has a lower incidence of MNM than countries in sub-Saharan Africa^4^, and a relatively low mortality index of 4.8%. There were, however, important differences between regions in Kenya. Although obstetric haemorrhage and hypertensive disorders of pregnancy were the commonest causes of mortality, fatality was similar across all major underlying causes of SMO. After haemorrhage and hypertensive disorders, abortive outcomes were the commonest cause of death.

While almost all patients with severe infection, postpartum haemorrhage and undergoing caesarean section received appropriate interventions, a large proportion of patients with other conditions did not receive the recommended evidence-based interventions. Most patients experienced their SMO before admission and majority of them were referred to the hospitals in our study from lower-level facilities. While patients with MNMs whose index pregnancy resulted in a live birth received a similar level of ANC by skilled providers (92%) as women with a live birth in the past five years in the most recent Kenyan DHS (96%)^12^, the proportion of MDs reporting any ANC was much lower (84%). The adapted Kenya criteria yielded almost 1.4 times the number of MNM cases than the WHO criteria did, and a large proportion of these patients were those who received laparotomies, and were additionally classified as having shock using an objective statistical algorithm.

To our knowledge, this is the first nationally representative, facility-based study describing the incidence and causes of MNM and MDs in Kenya. We collected data prospectively and utilized all the standardized criteria in the WHO near-miss tool, whilst adapting for local context, which allowed us to compare case yield from both criteria. This approach is helpful for allowing comparability with studies in other settings globally using the original WHO criteria, whilst allowing us to assess the performance of adaptations for the local context and compare them with successful adaptations identified in studies from other LMIC settings. That said, local adaptations of the MNM criteria often use additional clinical and management criteria, which may be defined or applied differently across hospitals and settings. Thus, applying these criteria comparably will require clear definitions and parameters to include cases. Overall, this kind of evidence will be helpful to generate a common tool for evaluating MNM that is more applicable to LMIC settings.

Some limitations of our study include its relatively short duration and the use of data from this period to generate annual estimates, which may not have accounted for seasonal trends in hospital admissions. Also, the study was conducted right after a national nurses and doctors strike across Kenya^18^, which most likely resulted in lower-than-typical caseloads^13,19^ and more MDs outside of hospitals around data collection^20–23^. This is most likely why our intra-hospital MMR was 10 times lower than the national MMR in the last DHS. Although, we invited private, county referral hospitals with substantial caseloads to participate in our study, they declined. We hypothesize that some of the caseload from public hospitals may have shifted to these facilities and potentially to other lower-level private facilities that were willing to admit women during the strike but not sampled in this study^24^. Non-participation from these facilities, which may have been managing severe obstetric cases, may also have contributed to the very low intra-hospital MMR and MNM ratio in this study. Additionally, despite our efforts to ensure high quality data collection, it is possible that we missed cases including those admitted to other wards or misclassified cases. Furthermore, because of the need to obtain consent from patients or their families before extracting clinical data, we had a higher proportion of MDs than MNMs from whom we could not obtain consent. Thus, our sample includes a higher proportion of eligible MNMs than maternal deaths.

The burden of MNM (0.7%) and SMO (0.8%) recorded in our study is similar to the estimates reported for Kenya from 20 hospitals within the 2013 WHO multi-country survey (0.4% and 0.7%, respectively)^25^. It is, however, lower than has been reported in a similar nationally representative study amongst referral facilities in Nigeria (1.6% and 2.7%, respectively), which is also a lower-middle income county in sub-Saharan Africa with a high MMR^4^. Kenya’s estimate is also lower than estimates of MNM for other countries in Africa with high MMR within the 2013 WHO multi-country survey^25^ and much lower than estimates reported in more recent smaller facility-based studies in Africa^8^. The intra-hospital MMR (36.2 per 100,000 live births) estimated in our study is also much lower than the Kenya estimate in the 2013 WHO multi-country study, which was 280 per 100,000 live births^25^. Additionally, the ratio of MNM to deaths in our study (20:1) is lower than the ratio reported in a recent study conducted in three counties in Western Kenya between 2014 and 2016 where there were 39 MNM cases for every one MD^26,27^. This study however differs from ours because it collected data on MNM and MDs from hospitals and within the community.

Our mortality index of 4.8% was much lower than the mortality index of 41% in Nigeria suggesting substantial differences in the quality of care provided for patients at referral facilities in both settings^4^. A mortality index below 20%^3^ and case fatality rates for direct obstetric complications of less than 1%^28^ are considered indicative of health systems that are managing severe cases optimally. Although Kenya’s indicators fall within the range of optimal care as described above, and were much lower than in other African countries^4,29^, over one-third of near-misses occurred during admission suggesting patients experience delays in receiving appropriate treatment in hospitals. There were also gaps in the coverage of evidence-based interventions for some conditions including the receipt of blood transfusion services for APH when it was required, prompt surgical intervention for uterine rupture, and magnesium sulphate for severe pre-eclampsia and eclampsia. Although we were unable to capture all the elements necessary to evaluate quality of care, these services are core components and process indicators of comprehensive emergency obstetric care (EmOC), which encompasses critical services to manage the commonest causes of maternal mortality at referral level hospitals^28^. These coverage gaps thus suggest that patients may receive substandard delivery care within Kenyan health facilities, which is similar to evidence from other studies that have evaluated quality of delivery care in Kenya more comprehensively^30,31^. Furthermore, the high proportion of SMOs that occurred before admission suggests that patients may be experiencing delays in receiving appropriate emergency care even when they have interacted with lower-level health facilities due to bottlenecks along the referral pathway^32,33^. A combination of individual/community level and health system interventions to address these delays are essential to improving patient’s obstetric outcomes^29^.

The volume of additional cases identified using the Kenyan adapted criteria is similar to that in other studies that used the original WHO criteria and compared with country-specific adaptations^8^. However, compared with other countries in sub-Saharan Africa that have examined the utility of the WHO near-miss criteria^8^, Kenyan public referral hospitals appear to have greater laboratory capability and were able to utilize a majority of the laboratory criteria included in the tool. Additionally, more patients needing massive blood transfusions in Kenya were able to obtain the WHO threshold of five or more units compared with other African countries like Tanzania^15^ and Ethiopia^34^ where this threshold was too high.

Although we provided training and visual aids containing study definitions for data collection, clinicians were likely to underreport cases of shock by selecting a checkbox when we compared their responses with an algorithm created from the most severe clinical signs recorded during admission. The published WHO data collection tool largely relies on clinicians selecting relevant categories of organ dysfunction (as a checkbox) to classify patients as MNM without collecting signs and symptoms for each possible category to verify the validity of this assessment^3,9^. To ensure that MNM cases are defined as objectively as possible, we recommend that future studies collect more detailed data on relevant clinical signs and symptoms to verify that clinicians’ assessments of organ dysfunction criteria aligns with the WHO definition.

Although Kenya recorded a low incidence of MNM during this period, coverage of essential EmOC interventions is suboptimal for many direct obstetric conditions. It is essential for the Kenyan government to strengthen the referral system and to provide essential EmOC interventions particularly blood transfusions, surgical services, oxytocin, and magnesium sulphate. Individual hospital administrators or regional Ministries of Health in Kenya may consider introducing facility audits of MNM events (in addition to MDs)^35^ as part of routine monitoring to enable them to identify institutional bottlenecks to providing high quality maternal care and to implement strategies to tackle the challenges identified. Future research should also explore the circumstances around the delays Kenyan women experience whilst accessing healthcare in more detail, evaluate the actual coverage of EmOC services at referral level facilities, examine the factors affecting availability and provision of essential interventions within the health system, and assess patient’s experiences of hospital care to provide a more balanced overview of quality.

## Supplementary information

Supplementary Information.

## Data Availability

The datasets generated and/or analysed during the current study are not publicly available. They belong to the Guttmacher Institute, USA and the African Population Health and Research Centre, Kenya but could be made available if a request is made to both parties organizations.
