# Supplementary material for: Incidence of maternal near-miss in Kenya in 2018: findings from a nationally representative cross-sectional study in 54 referral hospitals
Source: Sci Rep. 2020 Sep 16;10:15181. doi: 10.1038/s41598-020-72144-x (PMC7495416; doi:10.1038/s41598-020-72144-x)
Supplement: Supplementary file 1 — Supplementary Information. [file 41598_2020_72144_MOESM1_ESM.pdf]

Incidence of maternal near-miss in Kenya: findings from a nationally representative cross-sectional study in 54 referral hospitals

Onikepe Owolabi[1#*], Taylor Riley[1#], Kenneth Juma[2], Michael Mutua[2], Zoe H. Pleasure[1], Joshua Amo-Adjei[3], Martin Bangha[2]

1. Guttmacher Institute, Research Division, 125 Maiden Lane, 7th Floor, New York, New York, 10038, USA
2. African Population Health and Research Centre, Population Dynamics, Sexual and Reproductive Health Unit, Manga Close, Nairobi, Kenya
3. University of Cape Coast, Cape Coast, Ghana

[#] Contributed equally

*Corresponding author: Onikepe Owolabi

- Address: Guttmacher Institute, 125 Maiden Lane, 7th Floor, New York, New York, 10038, USA
- Phone: + 1 212-248-1111 ext. 2324
- Email: oowolabi@guttmacher.org

Supplementary Table S1: Sampling and response rate, by level of health facility and nationally, Kenya 2018

	**Facility level**				**Patient level**			
**Level of health facility**	Universe of health facilities	Sampling Proportion	Response rate	Total number of participating facilities	Total number of cases	Number of eligible cases	Response rate	Number of interviewed women
**Level 4**	424	11%	89%	41	269	266	100.0%	256
**Level 5**	16	94%	73%	11	314	311	99.4%	302
**Level 6**	2	100%	100%	2	144	141	99.3%	137
**Total**	442	14%	86%	54	727	718	99.6%	695

**All estimates are unweighted*

Supplementary Table S2. Study definitions

Potentially life-threatening condition (PLTC)*: These are an extensive category of clinical conditions, including diseases that can threaten a woman's life during pregnancy and labour and after termination of pregnancy.[1] We utilized all the conditions and interventions included in the WHO data collection tool,[2] and included additional criteria to ensure we incorporated all possible severe obstetric cases in the Kenyan context including septic abortion, prolonged obstructed labour, and uterine evacuation.*

Maternal near-miss (MNM)*: A woman who nearly died but survived a complication that occurred during pregnancy, childbirth or within 42 days of termination of pregnancy. We utilized the WHO maternal near-miss surveillance and assessment tool[1] and adapted it to include additional variables for clinical, laboratory and management-based criteria within each organ system category. These additions to the tool were based on work on validating the WHO approach in other LMICs[3] and based on feedback from our Kenyan collaborators about criteria commonly used in Kenyan referral hospitals that could also identify near-misses. The Kenya-adapted near-miss criteria is shown in Appendix Table 4 in comparison with the WHO maternal near-miss criteria.*

Maternal death (MD)*: The death of a woman while pregnant or within 42 days of termination of pregnancy, irrespective of the duration and the site of the pregnancy, from any cause related to or aggravated by the pregnancy or its management, but not from accidental or incidental causes (according to the tenth International Classification of Diseases [ICD-10].[4])*

Severe maternal outcome (SMO): A life threatening condition (i.e. organ dysfunction) including all maternal near-miss cases and maternal deaths.

Underlying cause of death: The disease or condition that initiated the morbid chain of events leading to death or the circumstances of the accident or violence that produced a fatal injury.

Live Birth (LB): Birth of an offspring, which breathes or shows evidence of life.

Severe maternal outcome ratio (SMOR): The number of women with life-threatening conditions (i.e. maternal death and near-misses) per 1000 live births. This indicator gives an estimate of the amount of care and resources that would be needed in an area or facility [SMOR = (MNM + MD)/LB].

Maternal near-miss ratio (MNMR): the number of maternal near-miss cases per 1,000 live births (MNMR = MNM/LB). Similar to the SMOR, this indicator gives an estimation of the amount of care and resources that would be needed in an area or facility.

Intra-hospital MMR: The number of maternal death that occurred in the hospital per 100,000 live births.

Mortality index (MI): The number of maternal deaths divided by the number of women with life-threatening conditions (i.e. maternal near-misses plus maternal deaths) expressed as a percentage [MI=MD/ (MNM+MD)]. The higher the index, the more the women with life-threatening conditions who die (indicative of low quality of comprehensive emergency care), whereas the lower the index the fewer women with life-threatening conditions who die (better quality of care).

References

1. Souza, J. P., Say, L., Pattinson, R. & Gulmezoglu, A. M. *Evaluating the quality of care for severe pregnancy complications: The WHO near-miss approach for maternal health*. 29 https://apps.who.int/iris/bitstream/handle/10665/44692/9789241502221_eng.pdf;jsessionid=E3D690DB8686A00D27FAC101222C7124?sequence=1 (2011).

2. Say, L., Souza, J. P. & Pattinson, R. C. Maternal near miss – towards a standard tool for monitoring quality of maternal health care. *Best Pract. Res. Clin. Obstet. Gynaecol.* **23**, 287–296 (2009).

3. Tura, A. K. *et al.* Applicability of the WHO maternal near miss tool in sub-Saharan Africa: a systematic review. *BMC Pregnancy Childbirth* **19**, 79 (2019).

4. *International Statistical Classification of Diseases and Related Health Problems 10th Revision*. https://icd.who.int/browse10/2016/en (2016).

Supplementary Table S3: Estimated annual incidence of maternal near-miss and other severe maternal outcome indicators in Kenya, by regions in 2018

	National	Nairobi & Central	Coast & North Eastern	Eastern	Nyanza & Western	Rift Valley
**Annual Number of Facility Caseloads**						
Total number of facility live births	708,459	196,189	57,247	101,044	168,523	185,455
Total number of facility deliveries	726,158	198,966	62,049	100,770	174,428	189,946
**Annual Number of Severe Maternal Outcomes**						
Number of maternal near-miss cases	5,116	1,199	467	437	1,264	1,749
Number of maternal deaths	256	100	16	81	8	51
**Overall Maternal Near-Miss Indicators**						
Severe maternal outcome (SMO) ratio (per 1,000 live births)	7.6	6.6	8.4	5.1	7.6	9.7
	(4.93 to 10.24)	(0.77 to 12.48)	(2.96 to 13.92)	(2.94 to 7.31)	(3.13 to 11.97)	(3.27 to 16.15)
Maternal near-miss incidence ratio (per 1,000 live births)	7.2	6.1	8.2	4.3	7.5	9.4
	(4.57 to 9.87)	(0.30 to 11.92)	(2.70 to 13.63)	(2.28 to 6.37)	(3.08 to 11.92)	(3.24 to 15.62)
Intra-hospital maternal mortality ratio (per 100,000 live births)	36.2	51.0	27.9	80.2	4.7	27.6
	(13.32 to 59.00)	(-16.13 , 118.08)	(22.52 to 33.38)	(-0.63 , 160.96)	(3.82 to 5.67)	(-18.78 , 73.99)
Mortality Index (MI)*	4.8%	7.7%	3.3%	15.6%	0.6%	2.8%
Maternal near-miss mortality ratio	20.0	12.0	29.2	5.4	158.1	34.2
**Hospital Access Indicators**	**%**	**%**	**%**	**%**	**%**	**%**
Severe maternal outcome occurred before admission in hospital	64%	70%	70%	55%	45%	73%
Severe maternal outcome occurred during stay in hospital	36%	30%	30%	45%	55%	27%
Severe maternal outcome occurred before admission, referred from other facilities	58%	59%	31%	39%	63%	66%
Mortality index: SMO occurred before admission	1.6%	4%	1.2%	1.7%	1.4%	0.3%

** Mortality index is calculated as: maternal deaths / (maternal near-miss + maternal deaths)*
***All cases are weighted*

Supplementary Table S4: Utilization of the WHO Near Miss Criteria adapted for Kenyan context								
	WHO Near Miss Criteria, by organ dysfunction criteria	WHO criteria*		Kenya criteria	Kenya-adapted criteria*		Women classified as MNM solely on the basis of criteria from Kenya adaptation	
		N	%		N	%	N	%
**MNM Total**		250	36%		360	52%	133	30%
Cardiovascular								
	Shock	94	14%	Shock**	109	16%	44	10%
	Cardiac arrest	5	1%	Cardiac arrest	5	1%		
Clinical Criteria				Severe anaemia (standalone < 4 Hb)	27	4%	9	2%
	Severe acidosis (pH < 7.1)	6	1%	Severe acidosis (pH < 7.1)	6	1%		
Laboratory markers	Severe hypofusion (Lactate > 5 mEq/mL)	1	0%	Severe hypofusion (Lactate > 5 mEq/mL)	1	0%		
Management based proxies	Use of continuous vasoactive drugs	12	2%	Use of continuous vasoactive drugs	12	2%		
	Cardio-pulmonary resuscitation	15	2%	Cardio-pulmonary resuscitation	15	2%		
Respiratory								
	Acute cyanosis	18	3%	Acute cyanosis	18	3%		
	Gasping	63	9%	Gasping	63	9%		
Clinical Criteria	Severe tachypnea (respiratory rate >40 breaths per min)	35	5%	Severe tachypnea (respiratory rate >40 breaths per min)	35	5%		
Laboratory markers	Oxygen saturation <90% for >=60 minutes)	44	6%	Oxygen saturation <90% for >=60 minutes	44	6%		
	PaO2/FiO2<200 mmHg	4	1%	PaO2/FiO2<200 mmHg	4	1%		
Management based proxies	Intubation and ventilation >60 mins not related to anaesthesia	11	2%	Intubation and ventilation >60 mins not related to anaesthesia	11	2%		
				Oxygen by mask or nasal tubes	64	9%	10	2%
Renal								
	Oliguria non-responsive to fluids or diuretics	44	6%	Oliguria non-responsive to fluids or diuretics	44	6%		
Clinical Criteria				Central oedema (puffy face, etc)	81	12%	22	5%
	Severe acute azotaemia (Creatinine ≥300μmol/l or ≥3.5 mg/dL)	32	5%	Severe acute azotaemia (Creatinine ≥300μmol/l or ≥3.5 mg/dL)	32	5%		
				Serum urea >8mmol	37	5%	7	2%
Laboratory markers				75% decrease in GFR calculated from 75mL/min per 1.73mm2	6	1%	0	0%
Management based proxies	Dialysis for acute renal failure	15	2%	Dialysis for acute renal failure	15	2%		
Coagulation/hematologic dysfunction								
	Failure to form clots	11	2%	Failure to form clots***	11	2%		
Clinical Criteria				Tendency to bleed	38	5%	13	3%
Laboratory markers	Severe thrombocytopenia (<50,000 platelets/ml)	15	2%	Severe thrombocytopenia (<50,000 platelets/ml)	15	2%		
				Abnormal liver function tests	21	3%	8	2%
Management based proxies	Transfusion of >= 5 units of blood	42	6%	Transfusion of >= 2 units of blood & HB level < 7	60	9%		
Hepatic dysfunction								
	Jaundice in the presence of pre-eclampsia	9	1%	Jaundice in the presence of pre-eclampsia	9	1%		
				Jaundice in the presence of sepsis	9	1%	1	0%
				Tender enlarged liver in PET	7	1%	4	1%
				Abnormal liver enzymes (AST <5 or >48 IU/L; ALT <7 or >55 IU/L)	32	5%	13	3%
Clinical Criteria				Abnormal liver enzymes with associated impaired consciousness	19	3%	5	1%
Laboratory markers	Severe acute hyperbilirubinemia (Bilirubin > 100μmol/l or >6 mg/dL)	16	2%	Severe acute hyperbilirubinemia (Bilirubin > 100μmol/l or >6 mg/dL)	16	2%		

Management based proxies				Admission for critical care	14	2%	1	0%
Neurologic dysfunction								
Clinical Criteria	Prolonged unconsciousness (lasting ≥ 12 hours)	39	6%	Prolonged unconsciousness (lasting ≥ 12 hours)	39	6%		
	Stroke	2	0%	Stroke	2	0%		
	Uncontrollable fits/status epilepticus	33	5%	Uncontrollable convulsions	33	5%		
	Total paralysis	0	0%	Total paralysis	0	0%		
Laboratory markers				EEG	8	1%	3	1%
				CT Scan	15	2%	2	0%
Management based proxies				Critical care	18	3%	2	0%
Uterine dysfunction								
Clinical Criteria				Paralytic ileus (Absent bowel sounds in a gaseous distended abdomen)	7	1%	1	0%
				PTE (pulmonary thromboembolism) showing the above respiratory symptoms and signs	3	0%	0	0%
Management based proxies	Hysterectomy following infection or haemorrhage	19	3%	Hysterectomy following infection or haemorrhage	19	3%		
				Laparotomy	45	6%	30	7%
**Incidence calculations********								
Maternal near-miss incidence ratio (per 1,000 live births)		4.7			7.6			
Severe maternal outcome ratio (per 1,000 live births)		4.9			7.9			

** Women can have multiple criteria*

*** Shock in WHO was defined based on clinicians simply selecting a woman as shock based on the training definition of persistent systolic blood pressure ≤80mm Hg alone or persistent systolic blood pressure ≤90mm Hg with a pulse rate at least 120 bpm, and requiring administration of any IV fluids and/or cold extremities, blurred vision. WHO in the Kenya adapted criteria was defined by using both clinicians assessment and an algorithm in statistical software to identify women with all the criteria for shock who were missed by clinicians.*

**** Failure to form clots defined as the absence of clotting from IV site >7 minutes*

***** Incidence is calculated using weighted cases and live births*

****** All cases are weighted*
